# Crohn’s disease: review and standardization of nomenclature

**DOI:** 10.1590/0100-3984.2022.0082-en

**Published:** 2023

**Authors:** Filipe C. B. Magalhães, Elissandra Melo Lima, Pedro Carpentieri-Primo, Miriam Menna Barreto, Rosana Souza Rodrigues, Daniella Braz Parente

**Affiliations:** 1 Universidade Federal do Rio de Janeiro (UFRJ), Rio de Janeiro, RJ, Brazil; 2 Grupo Fleury, Rio de Janeiro, RJ, Brazil; 3 Instituto D’Or de Pesquisa e Ensino (IDOR), Rio de Janeiro, RJ, Brazil

**Keywords:** Crohn disease, Tomography, X-ray computed/methods, Magnetic resonance imaging/methods, Magnetic resonance imaging, cine, Doença de Crohn, Tomografia computadorizada/métodos, Ressonância magnética/métodos, Imagem cinética por ressonância magnética

## Abstract

Crohn’s disease is an inflammatory bowel disease that can affect any segment of the gastrointestinal tract. It has a variable clinical course, with alternating periods of disease activity and remission. Because the incidence and prevalence of Crohn’s disease have been increasing, evaluation by imaging methods has become more important. The most widely used methods are computed tomography enterography, magnetic resonance enterography (as an elective examination), and contrast-enhanced computed tomography (in the context of emergency). Computed tomography enterography and magnetic resonance enterography are useful for diagnosis, follow-up, evaluation of complications, and prognosis. Both can be used in order to evaluate the small bowel loops and the associated mesenteric findings, as well as to evaluate other abdominal organs. They both also can detect signs of disease activity, fibrosis, penetrating disease, and complications. The interpretation of such changes is essential to the multidisciplinary approach, as is the standardization of the nomenclature employed in the reports. In this paper, we review and illustrate the imaging findings of Crohn’s disease, using the standardized nomenclature proposed in the multidisciplinary consensus statement issued by the Society of Abdominal Radiology, the Society of Pediatric Radiology, and the American Gastroenterology Association, with recommendations for descriptions, interpretations, and impressions related to those findings.

## Abstract (pt)

Doença de Crohn é uma doença inflamatória intestinal que pode acometer qualquer segmento do trato gastrointestinal, com curso clínico variado, alternando períodos de atividade de doença e remissão. Sua incidência e prevalência vêm aumentando, tornando a avaliação por métodos de imagem mais importante. Os métodos mais utilizados são enterotomografia computadorizada, enterorressonância magnética eletivamente e tomografia computadorizada com contraste no contexto da emergência. A enterotomografia computadorizada e a enterorressonância magnética são úteis para diagnóstico, seguimento, avaliação de complicações e para prognóstico. Avaliam as alças intestinais delgadas e os achados mesentéricos associados, além dos demais órgãos abdominais. Detectam sinais de atividade de doença, sinais de cronicidade e doença penetrante e complicações, importantes para a escolha do melhor tratamento. A interpretação dessas alterações é fundamental para que a equipe multidisciplinar trabalhe conjuntamente com compreensão da linguagem utilizada nos relatórios. Neste artigo, apresentamos uma revisão dos achados de imagem da doença de Crohn e ilustramos os possíveis achados dessa doença utilizando a nomenclatura padronizada proposta no consenso multidisciplinar composto pelas Sociedade de Radiologia Abdominal, Sociedade de Radiologia Pediátrica e a Associação Americana de Gastroenterologia, com recomendações de descrição, interpretação dos achados e recomendações de impressão.

## INTRODUCTION

Crohn’s disease is an inflammatory bowel disease that can affect any segment of the gastrointestinal tract, from the mouth to the anus. The clinical course is varied, alternating between periods of disease activity and remission. The incidence and prevalence of inflammatory bowel diseases have been increasing worldwide, and their importance has therefore been growing^(1)^. There has been a significant improvement in the survival of patients with these diseases, due to advances in treatment, including the use of biologic agents and improved surgical techniques^(2,3)^.

Computed tomography enterography (CTE) and magnetic resonance enterography (MRE) are imaging methods that have been used with ever increasing frequency. An oral preparation for distention of the bowel loops, which is essential for the evaluation of mural and perienteric alterations, is administered prior to CTE and MRE. The oral preparation is administered over a period of 40 min and contains neutral enteric contrast (3% polyethylene glycol or mannitol) diluted in 1.5 L of water^(4)^.

Because it requires only a short breath hold, CTE is a rapid test and is therefore well tolerated by uncooperative patients. In comparison with MRE, CTE has greater reproducibility, is more widely availability, provides greater spatial resolution, and is more easily evaluated by general radiologists. However, it has a lower capacity for tissue characterization and less capacity to differentiate between fibrosis and disease activity. In addition, CTE uses ionizing radiation, which can be harmful to patients with Crohn’s disease, who are typically young and undergo many tests throughout their lives^(4,5)^.

In comparison with CTE, MRE is a longer examination and requires greater patient cooperation because the protocol calls for multiple breath holds. In addition, it requires a 1.5-T or 3.0-T scanner to acquire good quality images. Therefore, MRE is less reproducible. Because of its higher cost and limited availability, MRI is, in general, less accessible than is CT. However, it is a technique that allows greater tissue resolution, making it the better method for differentiating between disease activity and fibrosis. Dynamic (cine) MRI allows the evaluation of peristalsis, increasing the accuracy of the method for detecting strictures, inflammation, and fibrosis. Furthermore, the use of multiple sequences provides a higher degree of confidence regarding enteric and perienteric alterations, with fewer false-positive and false-negative results. That also allows better characterization of fistulas and abscesses, as well as of other complications. Moreover, MRI does not use ionizing radiation and can be repeated multiple times, even in children^(4)^.

An evaluation employing the combination of CTE and ileocolonoscopy to observe Crohn’s disease activity has been shown to have a sensitivity of 84% and a specificity of 94%^(6)^. However, patients may not show changes in the segments evaluated by ileocolonoscopy, the results of which can be normal in cases in which the disease spares the colon, ileocecal valve, and distal ileum, making it even more important to complement it with other cross-sectional imaging methods that allow evaluation of the gastrointestinal tract as a whole^(6)^.

Endoscopy is also unable to assess the response to treatment in the submucosal layer, muscle, and adjacent structures. In addition to allowing an evaluation of the response to treatment in the intestinal wall, perienteric fat, and adjacent structures, MRE is also better than is CTE in differentiating between disease activity and fibrosis, which facilitates the individualization of treatment^(7)^. The main differences between CTE and MRE are presented in Table 1.

**Table 1:** Advantages and disadvantages of CTE and MRE.

CTE	MRE
Requires less patient cooperation	Requires considerable patient cooperation for a good quality examination
More accessible at different hospital levels	Less accessible
More affordable	More costly
Well visualized by generalist radiologists	Specialized radiologist required
Faster, tolerated better by emergency department patients	Longer, less tolerated by emergency department patients
Better spatial resolution; enables multiplanar reconstructions	Better tissue resolution; better characterization of active inflammation and fibrosis; peristalsis assessment (cine sequence)
Uses ionizing radiation	Does not use ionizing radiation

In the emergency department, patients with Crohn’s disease often present with intestinal distention typical of the underlying disease or its complications and do not tolerate the preparation for CTE. In such cases, CT examination with intravenous contrast administration is indicated. The intestinal distention already presented by the patient may be sufficient for the diagnosis. Contrast-enhanced CT is the imaging method most often used in the emergency department. In addition, CT can be used in order to evaluate the complications of Crohn’s disease, such as inflammatory masses, collections, fistulas, and perforations. It can also facilitate the differential diagnosis between abdominal pain related to Crohn’s disease activity and that related to other diagnoses, such as acute diverticulitis, acute appendicitis, acute cholecystitis, pancreatitis, mesenteric ischemia, and neoplasia. Furthermore, CT can be used in guiding interventional procedures, such as the drainage of fluid collections and intra-abdominal abscesses^(4,5,8)^.

There is currently a need to standardize the terminology used in radiology reports, to improve multidisciplinary understanding and the individualization of the treatment of Crohn’s disease. One valuable resource is the 2018 consensus statement on nomenclature authored by representatives of the Society of Abdominal Radiology, the Society of Pediatric Radiology, and the American Association of Gastroenterology, together with other experts on the subject^(9,10)^.

## SMALL INTESTINE IMAGING FINDINGS IN CROHN’S DISEASE DURING INFLAMMATORY ACTIVITY

### Inflammation-related findings

***Segmental mural hyperenhancement*** – This is defined as increased attenuation and increased signal in the various enteric (mural) layers on contrast-enhanced CT and MRI examinations, respectively. It is evaluated in the enteric phase (45–50 s after intravenous contrast administration) or in the venous phase (60–70 s after intravenous contrast administration), and the accuracy is similar in both phases^(11)^. Mural hyperenhancement can be classified as asymmetric, stratified, or homogeneous^(9,10,11)^.

**Figure 1:**
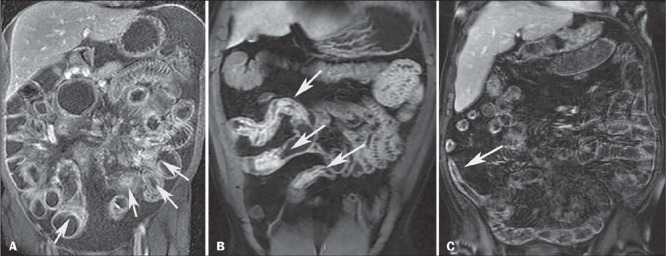
A: Contrast-enhanced coronal T1-weighted MRE sequence with fat suppression, showing asymmetric hyperenhancement preferentially involving the mesenteric border of the small bowel loops (arrows). B: Contrast-enhanced coronal T1-weighted MRE sequence with fat saturation in a patient with active inflammation, showing stratified (trilaminar) hyperenhancement of the small bowel loops (arrows), together with mural edema. C: Contrast-enhanced coronal T1-weighted MRE sequence with fat saturation, showing homogeneous hyperenhancement involving all layers of the small intestine (arrow), together with mild mural thickening, without upstream dilation.

• Asymmetric hyperenhancement is specific to Crohn’s disease and mainly involves the mesenteric border of the bowels (Figure 1A).

• Stratified hyperenhancement is defined as enhancement of the inner layer of the loop (bilaminar hyperenhancement) or of the inner and outer layers (trilaminar hyperenhancement; Figure 1B). The term “mucosal enhancement” should be avoided, because when enhancement of the inner layer is observed, the mucosa is no longer individualized on endoscopy. Stratified hyperenhancement can be associated with edema, granulation tissue, fat deposition, wall fibrosis, or inflammation.

• Homogeneous hyperenhancement is defined as enhancement involving all layers of the bowel uniformly (Figure 1C). It is less specific and can be due to fibrosis, intestinal ischemia, or collagen deposition.

**Figure 2:**
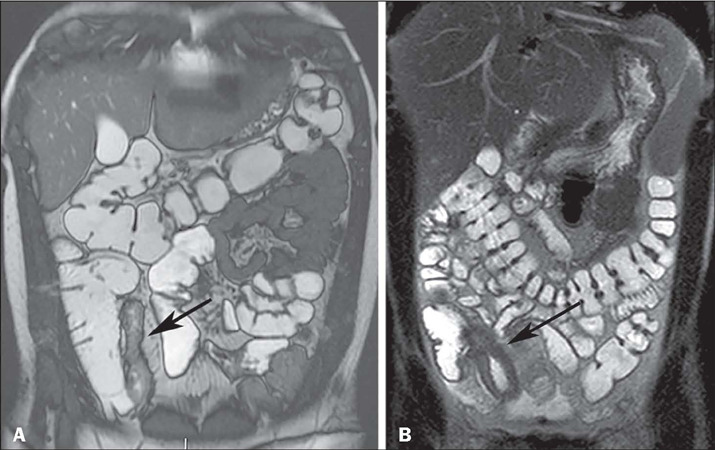
A: Coronal true fast imaging with steady-state precession MRE sequence showing moderate mural thickening of the terminal ileum (arrow), without dilatation of upstream bowel loops. B: Coronal T2-weighted MRE sequence showing marked mural thickening of the ileum and ileocecal valve (arrow).

***Segmental wall thickening*** – The thickest wall of the most inflamed segment should be measured, with good distension of the loop. Such thickening is classified as mild if the wall thickness is 3–5 mm (Figure 1C), moderate if it is 5–10 mm (Figure 2A) and severe if it is > 10 mm (Figure 2B). A wall thickness > 15 mm, especially if asymmetrical, is not an expected finding and should raise the suspicion of neoplasia^(9,10,11)^.

***Wall edema*** – This is defined as greater attenuation on CT and as high signal intensity on T2-weighted MRI sequences with or without fat suppression. On T2-weighted sequences without fat suppression, the differential diagnosis with fat deposition in the wall of a bowel loop, which also shows a hyperintense signal, is worthy of consideration. On T2-weighted sequences with fat suppression, the edema maintains the hyperintense signal and the fat shows a hypointense signal^(9,10,11)^.

***Stenosis*** – This is characterized as a ≥ 50% reduction in luminal diameter in comparison with that of the adjacent loop, together with unequivocal upstream dilation of the same loop (> 3 cm in caliber). Stenosis is most often seen in patients with active inflammation, although fibrosis and inflammation are often both present, in which case the inflammation leads to fibrosis and the fibrosis leads to inflammation, in a feedback loop. In cases of stenosis, penetrating disease should also be evaluated, because there is a mechanism of high pressure and inflammation, often causing fistulization proximal to the stenosis^(9,10,11)^.

**Figure 3:**
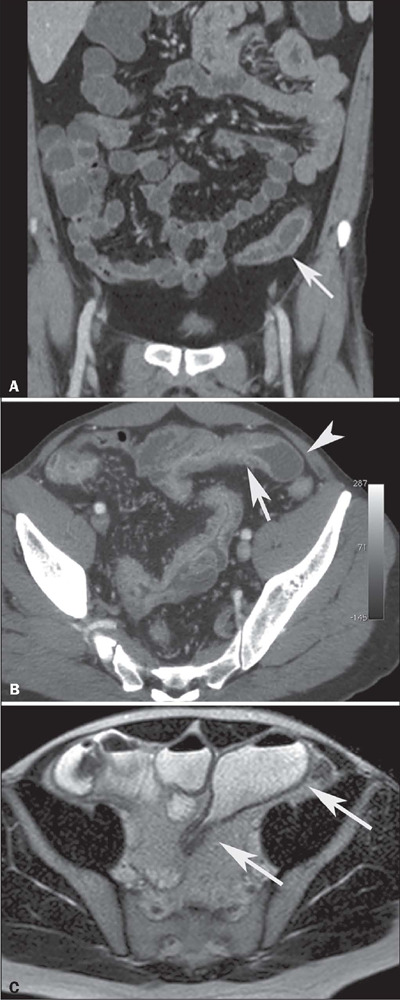
A: Coronal contrast-enhanced CT scan showing stenosis with mild mural thickening and stratified enhancement, without dilatation of upstream loops in the small intestine (arrow). B: Axial contrast-enhanced CT scan showing stenosis with moderate mural thickening and stratified enhancement (arrow), together with slight dilatation of upstream loops (arrowhead) in the small intestine. C: Axial T2-weighted MRE sequence showing stenosis with marked dilatation of upstream loops in the small intestine (arrows).

The extent of the stenotic segment and the caliber of the upstream dilatation should be described in the report. Figure 3A shows probable stenosis, with negligible (< 3 cm) dilatation, Figure 3B shows stenosis with mild (3–4 cm) dilatation, and Figure 3C shows stenosis with marked (≥ 4 cm) dilatation^(9,10,11)^.

***Ulceration*** – This is defined as a discontinuity of the inner wall of the bowel loop, with penetration of the lumi-nal contents into the wall of the loop. It is an indicator of severe disease activity^(9,10,11)^.

***Restricted diffusion*** – When there is inflammation, the wall of the bowel loops show restricted diffusion, increasing the sensitivity for detecting more subtle changes, which must be confirmed in the rest of the examination. However, luminal content and undistended loops can also show restricted diffusion. Therefore, diffusion-weighted sequences should always be interpreted together with the other sequences^(9,10,11)^.

***Outpouchings*** – These are defined as sac-like dilatations of the antimesenteric border of the bowel loop, resulting from acute or chronic inflammation with fibrosis at the mesenteric border^(9,10,11)^.

***Reduced loop motility*** – Cine MRE sequences can detect reduced peristalsis in bowel loops that are inflamed, fibrotic, or both. The reduction in peristalsis is proportional to the degree of inflammation and fibrosis^(9,10,11,12)^.

### Findings related to penetrating disease

**Figure 4:**
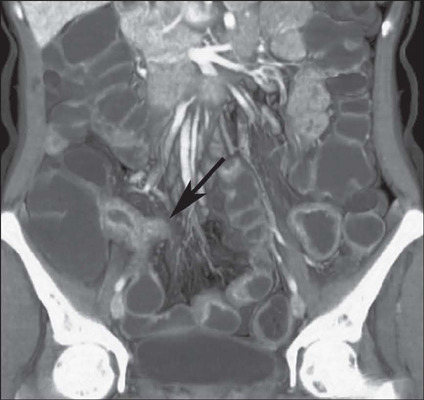
Coronal contrast-enhanced CTE, with maximum intensity projection, showing a sinus tract (arrow) in the terminal ileum with disease activity.

***Sinus tract*** – This is defined as a discontinuity of the bowel loop wall, extending to the perienteric fat, with a blind-ending, without reaching the surrounding structures or the skin^(9,10,11)^, as illustrated in Figure 4.

**Figure 5:**
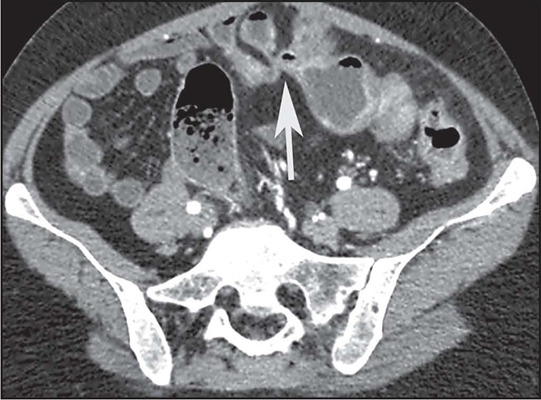
Axial contrast- enhanced CTE showing a simple enteroenteric fistula (arrow).

**Figure 6:**
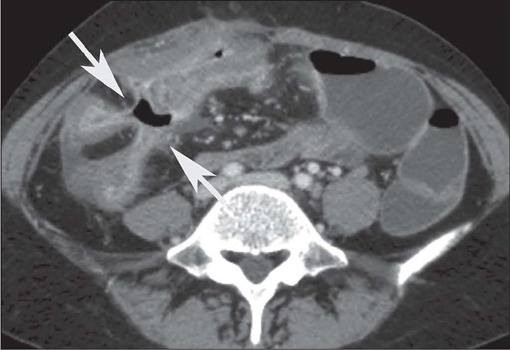
Axial contrast-enhanced CTE showing a complex fistula (arrows) between different bowel loops, in the shape of an asterisk.

***Simple and complex fistulas*** – A simple fistula is de-fined as a single extraenteric tract that connects a bowel loop with another loop or an adjacent organ (Figure 5). Complex fistulas are defined as multiple tracts connecting bowel loops with other loops or adjacent organs (Figure 6). Fistulas occur when there is active inflammation. The shape of a complex fistula can be described as a cloverleaf, asterisk, or star^(9,10,11)^.

***Inflammatory mass*** – This term refers to ill-defined inflammation of the fat, without a wall and without an organized liquid component, adjacent to a loop in which there is active inflammation^(9,10,11)^.

***Abscess*** – This is defined as a fluid collection with a well-defined wall that enhances on contrast-enhanced images and content that shows restricted diffusion, adjacent to a loop in which there is active inflammation^(9,10,11)^.

**Figure 7:**
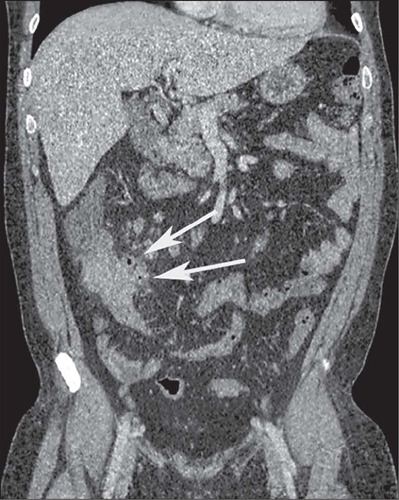
Coronal contrast-enhanced CTE showing small foci of gas near a bowel loop in which there was disease activity, consistent with a free perforation (arrows).

***Free perforation*** – Figure 7 shows an example of bowel loop perforation with active inflammation and free intraperitoneal air, which necessitates surgical evaluation^(9,10,11)^.

### Mesenteric findings related to Crohn’s disease

**Figure 8:**
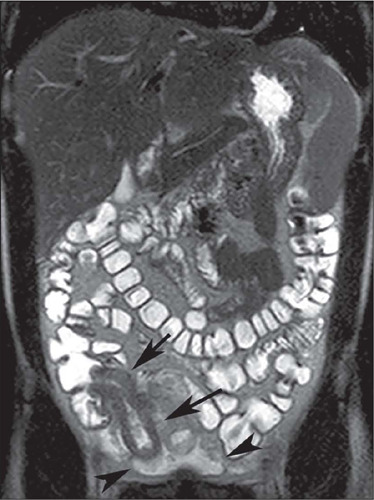
Coronal T2-weighted MRE with fat saturation, showing infiltration of the perienteric fat (arrows), together with a perienteric fluid collection (arrowheads), adjacent to the ileal loop, which showed disease activity.

***Perienteric edema or inflammation*** – Increased attenuation of perienteric fat on CTE or a hyperintense signal on T2-weighted MRE, adjacent to a loop in which there is active inflammation. Perienteric edema occurs when inflammation extends into the perienteric space^(9,10,11)^, as depicted in Figure 8.

**Figure 9:**
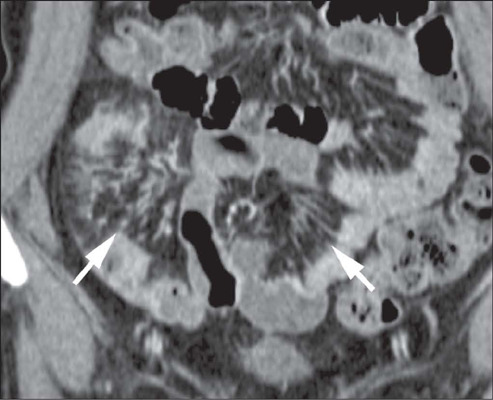
Coronal contrast-enhanced CTE showing engorged vasa recta (arrow) next to a bowel loop in which there was active disease.

***Engorged vasa recta*** – Increased blood supply and drainage of a small bowel loop segment (Figure 9), due to active inflammation, results in engorgement of the vasa recta, also known as the comb sign^(9,10,11)^.

**Figure 10:**
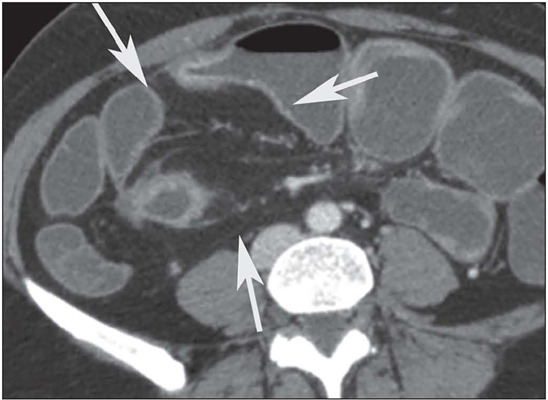
Axial contrast-enhanced CTE showing perienteric fibrofatty proliferation (arrows) in the ileal loop, due to long-standing disease.

***Fibrofatty proliferation*** – This is defined as hypertrophy of the perienteric fat adjacent to a bowel loop segment with long-term involvement by Crohn’s disease, detaching the loop from the neighboring structures (Figure 10). Such proliferation is a sign of chronicity^(9,10,11)^.

**Figure 11:**
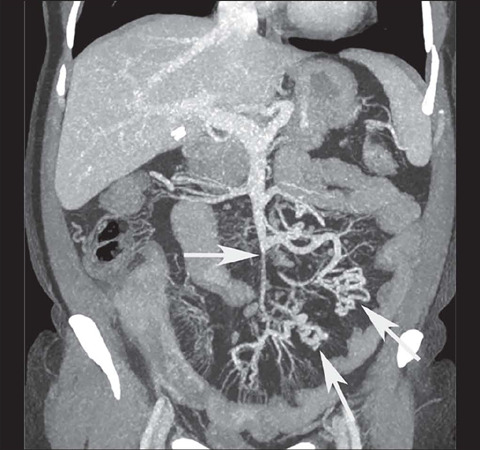
Coronal contrast-enhanced CTE, with maximum intensity projection, showing venous occlusion with reduced vessel caliber and multiple collateral mesenteric vessels with a chronic aspect (arrows).

***Chronic mesenteric venous occlusion*** – This occurs when there is occlusion of the collateral mesenteric vessels in segments of a bowel loop in which there is inflammation. In acute cases, in which the mesenteric vessels are typically distended by a thrombus, the term “thrombosis” should be used. In chronic cases, that term should not be used, because it could lead to the unnecessary use of anticoagulants. In such cases, the preferred term is “occlusion”. In cases of chronic mesenteric venous occlusion, the central mesenteric veins are narrowed (Figure 11)^(9,10,11)^.

***Reactive lymphadenopathy*** – This is defined as reactive lymph nodes, with a short-axis diameter of 1.0–1.5 cm, corresponding to a bowel loop in which there is active inflammation^(9,10,11)^.

### Extraintestinal manifestations of Crohn’s disease

As detailed in Table 2, the clinically relevant manifestations of Crohn’s disease include primary sclerosing cholangitis, pancreatitis, avascular necrosis, and sacroiliitis^(9,10,11)^. In its early stages, primary sclerosing cholangitis can often be identified on enterography as small focal dilatations of the intrahepatic bile ducts, and evaluation of the bile ducts by magnetic resonance cholangiopancreatography is indicated^(13)^. The main causes of pancreatitis in patients with Crohn’s disease are gallstones and pharmacological treatment, especially with azathioprine and mesalazine^(14)^, as well as with glucocorticoids and some other medications^(15)^. In such patients, pancreatitis typically has a mild or moderate clinical presentation and responds well after the end of treatment. Avascular necrosis mainly affects the femoral head, presenting clinically as hip pain. Sacroiliitis often manifests with low back pain as its main symptom, as confirmed by findings of (typically asymmetric) discrete erosions or even fusion of the sacroiliac joint^(16)^.

**Table 2:** Extraintestinal manifestations of Crohn’s disease.

Primary sclerosing cholangitis	Can affect the entire bile duct and presents with multiple strictures and intrahepatic dilatations or mural thickening of the extrahepatic bile duct, with contrast enhancement and without upstream dilation
Avascular necrosis	Focal sclerosis of the anterior portion of the femoral head, best visualized in the coronal plane
Sacroiliitis	Discrete bone erosions at sacroiliac joint fusion; high signal intensity on T2-weighted MRE sequences; sub-chondral edema; contrast enhancement; usually asymmetric but can affect the joint bilaterally
Pancreatitis	Consequent to cholelithiasis or pharmacological treatment
Cholelithiasis	Thought to result from inadequate reabsorption of bile salts^(16)^
Nephrolithiasis	Occurs in patients with diarrheal disease and ileal involvement; has a pathophysiological relationship with the formation of kidney stones^(17)^

## STRUCTURED REPORT

The radiology report should present the important information in a systematic manner (Table 3), in order to improve the quality and reproducibility of the communication with the multidisciplinary team. It is also important to provide a clear impression regarding inflammation, fibrosis, penetrating disease, and secondary involvement of distant structures^(9,10,11)^.

**Table 3:** Terms to be used in the impression section of the radiology report.

Inflammation
No signs of inflammation
Non specific enteric inflammation
Active inflammation without luminal stenosis
Active inflammation with luminal stenosis
Crohn's disease without signs of active inflammation
Stenosis
Stenosis with signs of active inflammation
Stenosis without signs of active inflammation
Penetrating disease
Sinus tract, simple fistula, inflammatory mass, free perforation, abscess

## TERMS TO AVOID IN THE FINAL REPORT

As established in the 2018 consensus, certain terms are no longer used (Table 4).

**Table 4:** Preferred use of terms.

Terms to be avoided	Preferred terms
Acute inflammation	Active inflammation
Fibrostenotic disease	Stenosis without active inflammation
Penetrating ulcer	Ulcer
Phlegmon	Inflammatory mass
Quiescent	Crohn’s disease without signs of active inflammation
Mesenteric venous thrombosis	Mesenteric venous occlusion
Mucous layer hyperenhancement	Bilaminar hyperenhancement

## CONCLUSION

In individuals with Crohn’s disease, CTE and MRE are valuable tools for diagnosis, assessment of the extent of the disease and its complications, as well as of associated conditions, facilitating the selection of the best clinical and surgical treatments, as well as the differential diagnoses. The structured radiology report allows the systematic evaluation of all structures and alterations to be described in Crohn’s disease and facilitates the follow-up of patients with the disease.
